# Development and Interpretability Analysis of Near-Infrared Spectroscopy Models for Fat and Protein Prediction in Foxtail Millet [*Setaria italica* (L.) Beauv.]

**DOI:** 10.3390/foods15040649

**Published:** 2026-02-11

**Authors:** Anqi Gao, Erhu Guo, Bin Wang, Dongxu Zhang, Kai Cheng, Xiaofu Wang, Aiying Zhang, Guoliang Wang

**Affiliations:** 1 Shanxi Houji Laboratory, Taiyuan 030031, China; gaq1137804400@163.com (A.G.); guoerhuo2003@163.com (E.G.); 2 Millet Research Institute, Shanxi Agricultural University, Changzhi 046000, China; wangbin_jczx@163.com (B.W.); gzszdx@163.com (D.Z.); kaikai6622@126.com (K.C.); 3 Key Laboratory of Minor Crop Germplasm Innovation and Molecular Breeding (Co-Construction by Ministry and Province), Ministry of Agriculture and Rural Affairs, Taiyuan 030801, China; 4 Department of Mechanical Engineering, Shanxi Institute of Engineering Technology, Yangquan 045000, China; 5 Department of Scientific Research Management, Shanxi Agricultural University, Taiyuan 030801, China; 19935150928@163.com

**Keywords:** foxtail millet, near-infrared spectroscopy, fat, protein, machine learning, interpretability

## Abstract

Foxtail millet is a nutritionally important cereal whose fat and protein content directly influence its nutritional quality and processing properties. To overcome the limitations of traditional detection methods, developing rapid, non-destructive, and interpretable models is essential. A total of 214 samples of the foxtail millet cultivar “Changnong No. 47” were used in this study. The Sparrow Search Algorithm was introduced to screen stable key wavelengths by statistically analyzing their selection frequency. Based on the selected wavelengths, quantitative models were constructed using Partial Least Squares Regression (PLS), Random Forest (RF), and Support Vector Machine. The SHapley Additive exPlanations method was employed to quantify the direction and magnitude of contributions of the key wavelengths within the model. Results show the selection of 13 key wavelengths for fat and 15 for protein. The RF model delivered the best prediction for fat content (R_P_^2^ = 0.797, RMSE_P_ = 0.218%, RPD_P_ = 2.219), while the PLS model performed best for protein content (R_P_^2^ = 0.695, RMSE_P_ = 0.268%, RPD_P_ = 1.811). The methodology established in this study can not only be applied to the rapid quality assessment of millet but also be extended to analyze the nutritional components of other grains.

## 1. Introduction

*Setaria italica* (L.) Beauv., commonly known as foxtail millet after hulling, is an ancient and nutritionally significant grain crop cultivated in arid and semi-arid regions of China and globally. As a hardy, drought-tolerant C4 crop with high water-use efficiency and contains substantial amounts of fat, protein, dietary fiber, vitamins, and minerals. This makes it a valuable and nutritionally dense food source [1]. Fat and protein are key indicators of its nutritional quality, processing suitability, and market value [2,3,4]. Fat acts as an important energy source, with its content and fatty acid profile directly influencing the flavor, aroma, and storage stability of foxtail millet [3]. Protein, as an essential macronutrient, is central to nutritional quality assessment. This assessment depends on its concentration and amino acid composition—especially essential amino acids like lysine [5]. Therefore, accurately determining the fat and protein content in foxtail millet is crucial for crop breeding, quality classification, processing improvement, and market oversight.

Traditional methods, like Soxhlet extraction (for fat) [6] and the Kjeldahl method (for protein) [7], are accurate but cumbersome, time-consuming. They require large amounts of organic solvents or highly corrosive reagents and are destructive. These limitations make them unsuitable for modern demands, such as large-scale screening in breeding, real-time monitoring, and rapid traceability in supply chains traceability. These applications require efficient and environmentally friendly detection techniques. Thus, developing rapid, non-destructive, and green detection technologies has emerged as a key focus in grain quality analysis.

Near-Infrared Spectroscopy (NIR) meets this demand well. Its works by detecting the overtone and combination vibrations generated when hydrogen-containing groups (C–H, O–H, N–H) absorb light at specific wavelengths [8]. Since different components have unique spectral fingerprints, their content can be quantitatively analyzed from the corresponding spectral data [9]. Compared with traditional methods, NIR technology offers distinct advantages. Sample pretreatment is simple or even unnecessary, enabling non-destructive or minimally destructive detection. Spectral acquisition takes only seconds, and when coupled with chemometric models, it allows for the simultaneous prediction of multiple components—making the process highly efficient and convenient. Furthermore, the entire procedure is reagent-free and environmentally friendly. Currently, NIR has been successfully applied to quality assessment in grains such as wheat, corn, and rice, as well as in oilseed crops including soybean and rapeseed.

NIR technology has been explored beneficially for detecting fat and protein in grains. Most studies use near-infrared spectrometers to collect spectra from grain powder or whole kernels. By applying machine learning algorithms, they build quantitative models linking spectral data to component content, achieving reasonable prediction accuracy. These works have confirmed the feasibility of using NIR technology for the compositional analysis of foxtail millet. Researchers have extensively studied fat and protein detection in various grains using different machine learning algorithms, producing many results. Kamboj et al. conducted research on fat content detection in wheat using near-infrared diffuse reflectance spectroscopy. They employed principal component analysis for feature extraction, selected nine core feature variables, and established a Partial Least Squares (PLS) prediction model, achieving accurate prediction of fat content in wheat [10]. Yu et al., focusing on protein content detection in maize, collected spectral information from intact maize kernels via near-infrared transmission spectroscopy and constructed a Support Vector Machine (SVM) model based on the extracted feature vectors [11]. Besides traditional algorithms, ensemble and deep learning methods are increasingly being used in this area. Yang et al. conducted research on rice protein detection [12]. They collected reflectance spectra of paddy rice and brown rice separately using near-infrared spectroscopy, and established protein content prediction models based on PLS, SVM, and Deep Neural Network (DNN). The results showed that the DNN model performed the best, demonstrating the advantages of deep learning algorithms. Yu et al. applied a Convolutional Neural Network (CNN) to the NIR detection of protein content in wheat [13]. Their model used preprocessed raw spectra as direct input, with convolutional and pooling layers automatically extracting features to build a prediction model. This approach demonstrated the effectiveness of deep learning in automatically capturing complex, high-level spectral features for quantitative analysis. In foxtail millet research, Bai et al. employed near-infrared diffuse reflectance spectroscopy combined with PLS to detect the contents of starch, protein, fat, pigments, and flavor compounds, and established a non-destructive detection model [14]. Although various machine learning algorithms combined with NIR have shown promising results in detecting grain fat and protein—advancing intelligent grain quality detection—most current models suffer from a “black box” problem: they lack interpretability. When these models map spectral features to component content, their internal decision processes, feature weights, and variable interactions remain unclear. For instance, in a CNN model, we cannot tell which spectral bands are crucial for predicting protein. Similarly, in an SVM, the mapping process of the kernel function is also difficult to explain through intuitive physical or chemical mechanisms. This black-box nature introduces significant risks to both research and practical application in grain component detection. The inability to pinpoint the core basis of a model’s decisions makes it difficult to deeply uncover the intrinsic correlation mechanisms between near-infrared spectra and the content of fat and protein in grains. This constrains theoretical innovation and optimization of the detection technology. When detection samples are in complex environments or vary greatly in cultivar, models may exhibit prediction deviations. However, the black box prevents these errors from being traced to their source, hindering model correction and affecting detection reliability and stability. Therefore, interpreting these machine learning black boxes is highly necessary and urgent. By conducting model interpretability research to clarify the internal decision-making logic and key influencing factors, we can not only enhance the credibility of detection models but also provide theoretical support for optimizing spectral preprocessing methods and improving feature extraction strategies. This will foster the deeper integration and sustainable development of NIR technology and machine learning in the field of grain quality detection.

Given this, this study focuses on rapidly and non-destructively detecting fat and protein in foxtail millet, with the aim of innovating methods and exploring the underlying mechanisms. The work includes the following steps:

- Growing and collecting representative foxtail millet samples under field conditions. Measuring reference values for fat and protein content, then analyzing their distributions and statistical characteristics.
- Introducing the Sparrow Search Algorithm to screen key wavelengths from foxtail millet’s near-infrared hyperspectral data. Through multiple independent runs and frequency-based statistics, establishing a stable wavelength selection strategy to obtain a robust set of informative key wavelengths.
- Building quantitative prediction models for fat and protein content based on the selected wavelengths. Identifying the best model by comparing performance metrics and analyzing reasons for differences in model performance.
- Using the SHAP interpretability framework to analyze the optimal model. This quantifies—from both global and local perspectives—the direction, magnitude, and mode of each key wavelength’s contribution to predictions, clarifying the model’s decision logic and improving its transparency and trustworthiness.

In summary, this study not only aims to construct high-performance NIR prediction models but also emphasizes innovative feature selection, deepened model interpretability, and elucidation of detection mechanisms. It aims to provide an efficient, reliable framework for foxtail millet quality assessment, offer a reference for detecting nutrients in other grains, and support the development of portable sensors based on key wavelengths. Ultimately, it aims to promote the application of non-destructive testing technology in breeding, quality control, and supply chain supervision.

## 2. Materials and Methods

### 2.1. Experimental Design and Material Collection

The field experiment for this study was conducted at the breeding base of the Foxtail Millet Research Institute, Shanxi Agricultural University (36°12′ N, 113°08′ E). The site has an elevation of 977 m and a warm temperate continental climate. The mean annual temperature is 10.2 °C, with around 185 frost-free days and about 550 mm of annual precipitation. These conditions provide excellent light and thermal resources, which fully meet the growth requirements of foxtail millet throughout its life cycle. The experimental field has cinnamon soil that is loose in texture and retains water and fertilizer well. Its suitable organic matter content creates a favorable environment for root development and nutrient uptake in foxtail millet. Stanley compound fertilizer (N–P_2_O_5_–K_2_O = 25–10–16, Stanley Agricultural Group Co., Ltd., Shandong, China) was applied as base fertilizer. With a reasonable ratio of nitrogen, phosphorus, and potassium, it supplies balanced nutrients for foxtail millet at the seedling stage and promotes robust early growth of the plants. The test variety used was “Changnong 47”. It was sown mechanically on 6 May 2025, with row spacing of 28 cm and plant spacing of 8 cm. At the jointing stage of the foxtail millet, urea was top-dressed at 245 kg/hm^2^ through a drip irrigation system. This integrated and precise water-fertilizer management helped improve nutrient use efficiency. The foxtail millet was harvested on 28 September 2025, once it reaching physiological maturity. We used a checkerboard sampling method to collect samples systematically across the planting area, covering different growth zones to minimize systematic error. A total of 214 samples were collected, each weighing 250 g. The sample size and randomness met the requirements for later lab analysis. After harvesting, the samples were sun-dried naturally to below 13% moisture and then mechanically hulled to obtain clean, intact millet kernels.

### 2.2. NIR Hyperspectral Data Acquisition

The foxtail millet was evenly placed into a container measuring 5 cm in diameter and 3 cm in depth, and the surface was leveled and lightly compacted. A push-broom hyperspectral imaging system (Headwall Photonics, Bolton, MA, USA) was used to collect NIR hyperspectral data. The system covers a spectral range of 900–1700 nm with 172 bands, and has a spectral resolution of 4.715 nm. The distance from the lens to the sample surface was fixed at 280 mm, with the push-broom scanning speed set to 2.721 mm/s. Data calibration was performed using a white reference standard and a dark current reference obtained by covering the lens, followed by final calculation according to Equation (1).

(1) $$R=\frac{R_{0}-R_{b}}{R_{w}-R_{b}}$$

where *R* is the calibrated image; *R*_0_ is the original raw image; *R_w_* is the white reference calibration image (reflectance ≈ 99.9%); and *R_b_* is the dark reference calibration image (reflectance ≈ 0%).

To minimize interference from instrument dark current at the spectral edges (<950 nm and >1650 nm) and to improve both model stability and spectral feature validity, the wavelength range of 950–1650 nm was chosen as the effective modeling interval, which includes 148 wavelengths. This spectral region is well-suited for developing hyperspectral quantitative detection models for fat and protein content in foxtail millet. For each prepared sample, spectral data were acquired three times consecutively and averaged to ensure representative data.

### 2.3. Determination of Fat and Protein Content

After hyperspectral data acquisition, the foxtail millet was homogenized, ground into powder, and passed through a 40-mesh sieve.

The Soxhlet extraction method was used to determine the fat content [6]. Approximately 2 g of the ground and sieved foxtail millet powder was weighed, and continuously extracted for 6–8 h in a Soxhlet apparatus using petroleum ether (boiling point range 30–60 °C) as the solvent. After extraction, the solvent was recovered, and the extraction flask was dried to constant weight in an oven at 105 °C. The fat content was calculated using Equation (2).

(2) $$Fat=\frac{P}{m}\times 100\%$$

where *P* denotes the weight gain of the extraction flask after extraction (g); *m* represents the sample mass (g).

Protein content was measured using GB 5009.5–2025 [7]. Approximately 0.5 g of the ground and sieved foxtail millet powder was weighed and transferred to a Kjeldahl flask along with a catalyst mixture (potassium sulfate and copper sulfate). Concentrated sulfuric acid was added for digestion until the solution became clear. The digested solution was then alkalized and distilled, and the released ammonia was absorbed in a boric acid solution. Finally, titration was performed using a standardized hydrochloric acid solution. The protein content was calculated using Equation (3).

(3) $$Pr=\frac{\left(V_{1}-V_{0}\right)\times C\times 0.014\times 5.83}{m}\times 100\%$$

where *Pr* represents protein content (%); *V*_1_ denotes the volume of hydrochloric acid consumed in titrating the sample (mL); *V*_0_ represents the volume of hydrochloric acid consumed in the blank titration (mL); *C* is the concentration of the standardized hydrochloric acid solution (mol/L); *m* stands for the mass of the sample (g); and 5.83 is the conversion factor for converting nitrogen content to protein content in foxtail millet.

For each sample, the fat and protein contents were determined in triplicate, and the final result was taken as the average of the three measurements.

### 2.4. Spectral Data Preprocessing

During the acquisition of hyperspectral images from foxtail millet samples using the hyperspectral imaging system, interference factors such as instrument noise, environmental noise, and surface scattering are inevitable. Preprocessing the raw spectral data is therefore essential. Its main goals are to remove instrumental and environmental noise, reduce surface scattering effects, and improve the reliability of the spectral signals.

Savitzky-Golay (S-G) smoothing [15,16] is a nonlinear filtering method based on local polynomial fitting. Its primary function is to filter out high-frequency noise from spectral curves. Within a predefined local window width, the method fits the spectral data inside the window using a polynomial function via the least squares method. This achieves smoothing and optimization of the original spectra, reducing noise interference while preserving the effective spectral information.

Standard Normal Variate (SNV) [17,18] is a correction method based on single-sample statistics, focusing on addressing multiplicative scattering effects caused by factors such as particle size differences, varying surface gloss, and uneven distribution. It also suppresses the influence of background interference on reflectance spectra. Its core concept involves centering and scaling each individual spectrum separately. This process transforms the spectral data of each sample to approximately follow a standard normal distribution, effectively improving the overall consistency of the spectral data.

To ensure that the model built from preprocessed spectral data has strong generalization capability and reliable prediction performance, the sample set was randomly divided using the Hold-Out method [5,19] during model development and validation. The samples were randomly allocated into training and prediction sets at a 3:1 ratio. The training set was used for model training, where parameters were optimized and structural adjustments made, providing fundamental support for the model to learn the inherent laws of spectral data. The prediction set, which was kept separate from the training process, was used to evaluate the model’s prediction accuracy on unseen samples. This approach helped ensure an objective and accurate assessment of model performance.

### 2.5. Key Wavelength Selection

Hyperspectral imaging captures reflectance information across dozens to hundreds of contiguous narrow bands. This provides rich material characterization, but also introduces challenges like high data dimensionality, significant information redundancy, strong multicollinearity, and noise interference. Using the full spectrum directly can greatly increase model complexity and computational cost. It may also lead to model overfitting by including irrelevant or noisy variables, which reduces generalization capability and prediction reliability. Therefore, key wavelength selection is a critical step. It involves extracting a subset of wavelengths from the original spectra that are most relevant to the target attribute and richest in information. This process helps simplify the model, improve prediction accuracy, and enhance model interpretability.

To achieve this objective, this study introduces the Sparrow Search Algorithm (SSA) [20,21,22] as an efficient optimization tool. SSA is a meta-heuristic optimization algorithm inspired by the foraging and anti-predation behaviors of sparrow populations. By simulating the division and cooperation among three types of individuals—discoverers, followers, and sentinels—it maintains a good balance between global exploration and local exploitation, effectively avoiding premature convergence. Characterized by its fast convergence and strong search capability, SSA can efficiently locate high-performing feature subsets within the vast feature combination space.

Step 1: Randomly generate an initial population $S=\left[S1,\;S2,\;\cdots ,\;SD\right]\in \left\{0,1\right\}$ consisting of binary vectors. Each individual represents a candidate feature subset *D*, where *D* denotes the total number of original features. In this encoding, *s_j_* = 1 indicates that the *j*-th feature is selected, and *s_j_* = 0 indicates it is not selected. Each subset is constrained to contain at least one selected feature.

Step 2: For each feature subset within the population, its fitness is calculated using Equation (4). The fitness value is defined as the mean squared error (*MSE*) of the model when trained and evaluated using that specific subset of features. A lower MSE corresponds to a better fitness score.

(4) $$\mathrm{Fitness}(s)=MSE(s)=\frac{1}{N}\sum_{i=1}^{N}{\left(yi-\hat{y}i(s)\right)}^{2}$$

where *N* denotes the number of training samples; *y_i_* is the actual value of the *i*-th sample; and ${\hat{y}}_{i}(s)$ is the predicted value for the *i*-th sample generated by the regression model trained on the feature subset defined by vector *s*. Specifically, the feature columns are first extracted from the original data matrix according to *s* to obtain the subset data, on which the model is then trained and used for prediction.

Step 3: Iterative Optimization. For the top-ranked discoverers based on fitness, a portion of their features were randomly mutated with a certain probability to generate new feature subsets. If the new subset yielded a lower MSE, it replaced the original solution. Followers randomly selected a discoverer and, based on its feature subset, performed feature flipping with a high probability to create a new subset. Similarly, replacement occurred if the new subset had a lower MSE. For the sentinels with the poorest fitness, their feature vectors were completely reinitialized randomly to help the population escape potential local optima.

Step 4: Selection and Recording. After each generation, the global best feature subset and its corresponding lowest MSE were recorded and updated.

Step 5: Output Results. Upon completion of the iterations, the algorithm outputs the indices of the features in the best-performing subset and the lowest MSE achieved when modeling with this subset.

By running the SSA independently multiple times (a total of 50 runs), the selection frequency of each feature was statistically analyzed. Features with an occurrence probability greater than 50% were selected as the high-reliability candidate set. Three types of candidate sets were constructed: first, all features with a probability >50%; second, the top N features sorted by selection probability (where N ranges from 10 to 50 with a step size of 5); third, feature sets filtered by different probability thresholds (0.5, 0.6, 0.7, 0.8, 0.9), with each set containing at least 5 features. Subsequently, 5-fold cross-validation and PLS regression models were employed to evaluate the performance of each candidate set. Taking MSE as the primary evaluation metric, the average MSE and Root Mean Squared Error (RMSE) for each candidate set across the cross-validation folds were calculated. Ultimately, by comparing the MSE values of all candidate sets, the set with the smallest MSE was selected as the optimal feature set. The objective was to identify, from multiple dimensions, an optimal feature subset that ensures both model prediction accuracy and a reasonable number of features, thereby guaranteeing that the selected features possess both representativeness and generalization capability. This process, through multiple runs and statistical evaluation, effectively mitigates the impact of algorithmic randomness and achieves data-driven optimal feature selection. The main parameter settings in its SSA were as follows: a population size of 100, an iteration count of 500, a discoverer proportion of 0.7, a vigilance proportion of 0.2, and a safety threshold of 0.8.

### 2.6. Detection Model Construction and Interpretability

The core of PLS lies in its ability to simultaneously perform dimensionality reduction and regression by iteratively extracting latent variables from both the independent variable matrix X and the dependent variable Y [5,10,12,14]. It maximizes the covariance between these latent variables and Y, thereby preserving key information while filtering out noise. Ultimately, a regression model is built using a small number of latent variables, effectively addressing issues like the curse of dimensionality and multicollinearity. This makes it particularly well-suited for scenarios such as spectral data analysis. The number of latent variables—its primary parameter—was selected via 5-fold cross-validation.

RF is an ensemble learning algorithm based on decision trees [23,24,25]. Its core mechanism involves building a large number of diverse trees through dual randomness (bootstrap sampling of the training set and random selection of splitting features), and then integrating the results by averaging their predictions. This mechanism effectively suppresses overfitting, captures complex non-linear relationships, and can output feature importance rankings, balancing both predictive performance and model interpretability. The main parameters were configured as follows: the number of trees was set to 100, the minimum leaf size was 5, and the maximum depth was determined based on the minimum leaf node count, thus controlling model complexity.

SVM is based on the principle of structural risk minimization, aiming to identify the optimal decision boundary and maximize the classification margin to enhance generalization capability [5,11,12]. For linearly inseparable problems, SVM employs kernel functions to map data into a higher-dimensional space for linear separation. The model ultimately relies on only a few support vectors for decision-making, exhibiting sparsity and robustness. It excels in handling small-sample, high-dimensional nonlinear problems but is relatively sensitive to the selection of kernel functions and parameters. The main hyperparameters were optimized using a random search strategy.

SHAP is an interpretability framework based on game theory [26,27,28]. It explains model predictions by calculating the contribution of each feature to an individual prediction. SHAP values possess an additive property, allowing for a clear illustration of whether a feature has a positive or negative impact on the prediction outcome. This framework supports both local explanations (for single predictions) and global explanations (for overall model behavior). It effectively unveils the decision-making logic of black-box models, thereby enhancing their trustworthiness and practical utility.

Model performance was evaluated using the coefficient of determination (R^2^), RMSE, and relative percent deviation (RPD). An RPD > 2.0 indicates good to excellent predictive performance; an RPD between 1.4 and 2.0 suggests the model has moderate predictive capability; and an RPD < 1.4 signifies that the model lacks sufficient predictive accuracy [5,17,20]. All data processing and analyses were performed using MATLAB software (Version 2023b, MathWorks, Natick, MA, USA).

## 3. Results and Analysis

### 3.1. Analysis of Variance in Fat and Protein Content in Foxtail Millet

**Figure 1:**
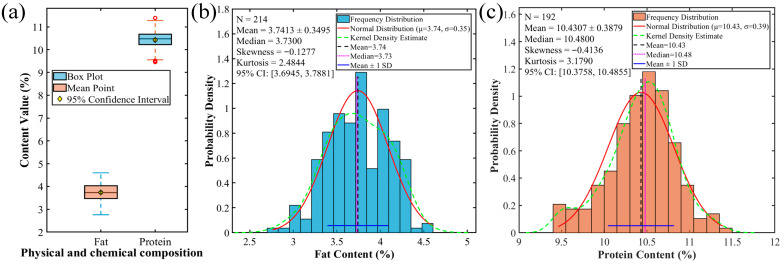
Distribution characteristics and statistical comparison of fat and protein content in foxtail millet samples. (**a**) Box plots and 95% confidence intervals for fat and protein content; (**b**) Distribution fitting plot for fat content; (**c**) Distribution fitting plot for protein content.

In this study, the interquartile range method was employed for outlier detection and processing of the fat and protein content data from 214 foxtail millet samples, as shown in Figure 1a. The results indicated that no outliers were detected in the fat content data, so all samples were kept in subsequent analyses. However, 22 outliers (accounting for 10.28%) were identified in the protein content data. After removing these, 192 valid samples were retained for further analysis. Statistical analysis of the fat content, as shown in Figure 1b, revealed a mean value of 3.74% with a standard deviation of 0.35%, yielding a coefficient of variation (CV) of 9.34%, which indicates a moderate level of dispersion. The data distribution exhibited a slight negative skew (skewness = −0.13) and low kurtosis (kurtosis = 2.48), with a 95% confidence interval of [3.69%, 3.79%]. The fat content was concentrated within the range of 3.30% to 4.21%, with half of the samples exceeding 3.73%. Based on the box plot and distribution fitting, the fat content was approximately normal, symmetric, and concentrated, and the mean estimate was precise (narrow confidence interval). For protein content, as shown in Figure 1c, the mean was 10.43%, much higher than that of fat. Its CV was only 3.72%, markedly lower than that of fat, suggesting greater stability in protein content across different samples. The distribution showed moderate negative skew (skewness = −0.41) and relatively high kurtosis (kurtosis = 3.18), with a 95% confidence interval of [10.38%, 10.49%]. The median value (10.48%) was slightly higher than the mean, confirming a slight shift toward lower values. Comparative analysis revealed significant differences between the two components. The mean difference between fat and protein content reached 6.69%, with a CV ratio of 2.51:1. This indicates that fat content is not only lower in absolute value but also exhibits greater variability among samples. The 95% confidence intervals for both indicators are relatively narrow, confirming that the estimation of population means based on the current sample set possesses high precision and reliability. These findings provide crucial data support for constructing a quality evaluation system for foxtail millet and for optimizing variety breeding strategies.

### 3.2. Spectral Response and Preprocessing of Foxtail Millet

**Figure 2:**
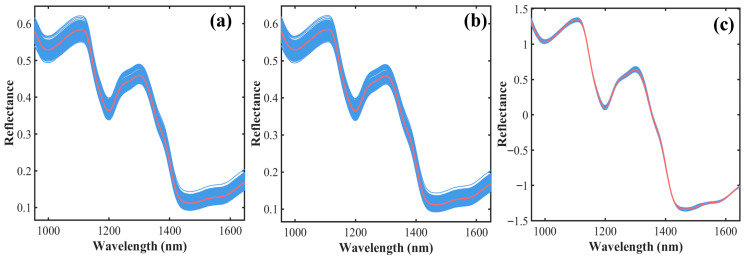
Spectral preprocessing of foxtail millet. (**a**) Raw spectral response; (**b**) Result after S-G preprocessing; (**c**) Result after S-G and SNV preprocessing. (Note: The blue lines represent the average spectral curves of each sample; the red line represents the average spectral curve of all the samples).

Figure 2a presents the NIR reflectance spectra of foxtail millet. The reflectance exhibited regular fluctuations across different wavelength ranges. Overall, the reflectance showed a decreasing trend around 950–1000 nm, 1100–1200 nm, and 1300–1450 nm. This indicates stronger absorption of light energy by the samples in these bands, which primarily reflects the combined molecular vibrational information of major components in foxtail millet, such as fat, protein, starch, and moisture [29]. In contrast, within the wavelength intervals of 1000–1100 nm, 1200–1300 nm, and 1450–1650 nm, reflectance generally exhibited an increasing trend. This suggests relatively weaker absorption in these bands, which may be related to enhanced scattering properties of the millet or transitions between absorption peaks of different components [30].

Figure 2b demonstrates the spectral outcome after S-G smoothing. This method effectively suppressed high-frequency random noise in the original spectra, yielding a smoother signal curve. Concurrently, it preserved the shapes of key absorption peaks and valleys without inducing significant peak shifts or broadening, which is advantageous for subsequent spectral feature extraction and modeling analysis. Figure 2c further illustrates the correction effect of SNV processing on the spectra. After SNV treatment, spectral baseline drift and intensity variations caused by physical factors were markedly reduced, enhancing spectral comparability across different samples. Consequently, the spectral features corresponding to chemical components such as fat and protein in foxtail millet were more prominently highlighted. Using the preprocessed spectral data combined with fat and protein content, the dataset was partitioned into training and prediction sets via the hold-out method. For fat, the training and prediction sets comprised 161 and 53 samples, respectively. Correspondingly, for protein, the training and prediction sets consisted of 144 and 48 samples, respectively.

### 3.3. Key Wavelength Selection and Model Construction for Fat and Protein

**Figure 3:**
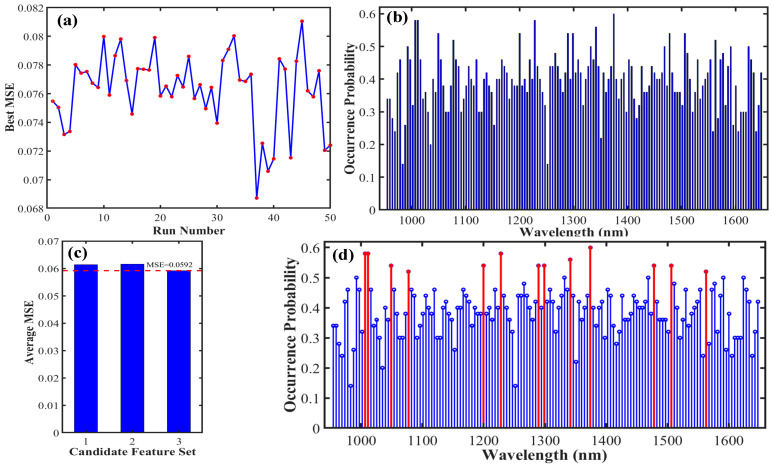
Key wavelength screening results for fat content in foxtail millet. (**a**) Optimal MSE from each of the 50 independent SSA runs (The red dots indicate the minimum MSE value for each instance); (**b**) Frequency statistics of the screened wavelengths; (**c**) MSE of the candidate sets (The area marked by the red dotted line represents the minimum MSE value); (**d**) Screened optimal key wavelengths (wavelengths marked by the red line).

To accurately identify key wavelengths associated with the fat content of foxtail millet, this study employed the SSA for optimal feature wavelength selection. The algorithm was independently performed 50 times. In each run, a prediction model was built based on the selected wavelength subset, with the model’s MSE serving as the evaluation metric. The SSA effectively identified wavelength combinations that resulted in lower model errors. The wavelength subset corresponding to the optimal MSE value was identified as the best screening result for that individual run, as illustrated in Figure 3a. To further extract stable and reliable key wavelengths from multiple runs, we conducted a statistical analysis of the occurrence frequency of each selected wavelength across the 50 runs, with the results shown in Figure 3b. The results showed that certain wavelengths were chosen much more frequently than others, indicating stronger relevance and stability for predicting fat content. Based on this frequency distribution, a preliminary candidate set of key wavelengths was constructed using a threshold of 50% or higher occurrence frequency. This set encompasses important spectral regions that were repeatedly validated by the algorithm. To further identify the most representative key wavelengths, a strategy involving the construction of three types of candidate sets was proposed. In addition to the high-frequency wavelength set mentioned above, this strategy also considered a core wavelength set corresponding to the highest frequency points and an optimized wavelength set selected through backward screening based on model errors. By comprehensively comparing the predictive performance of models built from each candidate set and adhering to the principle of MSE minimization, the optimal key wavelength set was ultimately determined, as shown in Figure 3c,d. This optimal set ensured model accuracy while achieving an effective reduction in the number of wavelengths, significantly lowering the dimensionality of the spectral data. The results indicated that 13 key wavelengths most closely associated with the fat content of foxtail millet were identified: 1003.51, 1011.22, 1048.94, 1077.23, 1199.81, 1228.10, 1289.39, 1298.82, 1341.25, 1374.25, 1477.98, 1506.26, and 1526.84 nm. To validate the predictive capability and generalizability of the selected key wavelength set, we constructed PLS, RF, and SVM models, respectively. All models demonstrated favorable performance on the prediction set based on the key wavelength set, as indicated by their R^2^ values. Among these, the RF model exhibited the best overall predictive performance (R_P_^2^ = 0.797, RMSE_P_ = 0.218%, RPD_P_ = 2.219), highlighting its advantage in handling nonlinear spectral relationships. The PLS and SVM models also delivered robust results, with prediction set R^2^ values of 0.742 and 0.737, and RPD values of 1.969 and 1.950, respectively. This indicates that the linear approach (PLS) and the kernel-based nonlinear method (SVM) remain effective, though slightly less accurate than RF for this specific component. A detailed comparison of the evaluation metrics for each model is presented in Table 1. In summary, the wavelength selection conducted via the SSA, combined with multi-strategy candidate set optimization, enables the stable and effective identification of key spectral features associated with fat content in foxtail millet.

**Figure 4:**
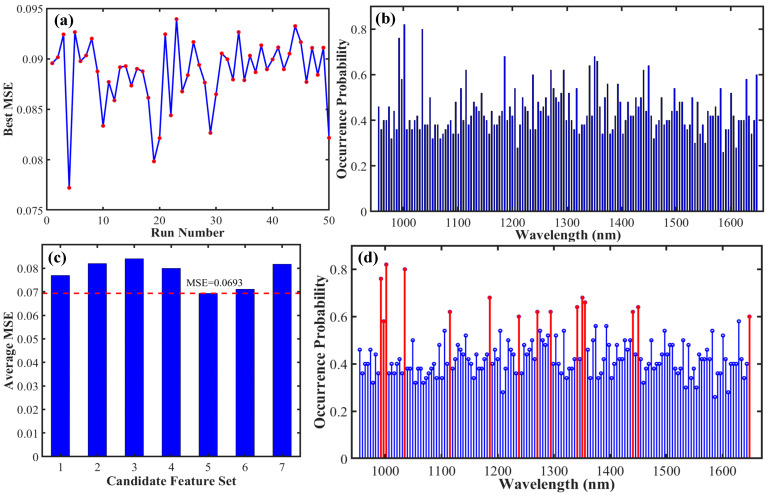
Key wavelength screening results for protein content in foxtail millet. (**a**) Optimal MSE from each of the 50 independent SSA runs (The red dots indicate the minimum MSE value for each instance); (**b**) Frequency statistics of the screened wavelengths; (**c**) MSE of the candidate sets (The area marked by the red dotted line represents the minimum MSE value); (**d**) Screened optimal key wavelengths (wavelengths marked by the red line).

To precisely identify key wavelengths associated with the protein content of foxtail millet, the methodology and processes described in Figure 4a–d were consistent with those outlined for fat content in Figure 3a–d. This optimal set ensured model accuracy while achieving an effective reduction in the number of wavelengths. The results indicated that 15 key wavelengths most closely related to the protein content of foxtail millet were identified: 992.363, 997.077, 1001.79, 1034.8, 1114.94, 1185.67, 1237.53, 1270.53, 1294.1, 1341.25, 1350.68, 1355.39, 1440.26, 1449.69, and 1647.71 nm. Among these, the PLS model demonstrated the best overall predictive performance (R_P_^2^ = 0.695, RMSE_P_ = 0.268%, RPD_P_ = 1.811). In contrast, the nonlinear models, RF and SVM, exhibited significantly lower predictive accuracy, with R_P_^2^ values of only 0.472 and 0.538, respectively. A detailed comparison of the evaluation metrics for all models is provided in Table 1.

### 3.4. Model Interpretability Analysis

**Figure 5:**
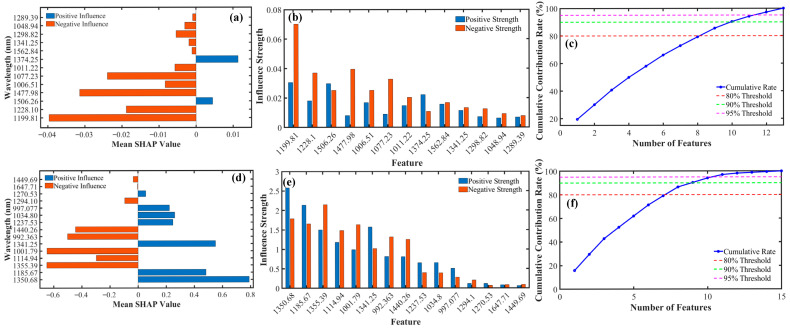
SHAP interpretability results for key wavelengths in the optimal models for fat and protein. (**a**,**d**) Mean SHAP values of the key wavelengths; (**b**,**e**) Positive/Negative directional effects of the key wavelengths; (**c**,**f**) Contribution rates of the key wavelengths. ((**a**–**c**): Fat; (**d**–**f**): Protein).

To further elucidate the internal decision-making mechanism of the RF prediction model for fat content in foxtail millet—constructed based on key wavelengths—and to enhance the model’s interpretability and credibility, this study introduced the SHAP method for an in-depth analysis of this black-box model. By comprehensively averaging the SHAP values across all samples, the overall impact trend of each wavelength on the model output was obtained, as shown in Figure 5a. The analysis revealed that within the final set of selected key wavelengths, only two wavelengths (1374.25 nm and 1506.26 nm) had positive average SHAP values. This indicates that, overall, they exert a positive driving effect in the model—meaning an increase in their spectral reflectance intensity tends to elevate the predicted value for fat content. In contrast, the average SHAP values for all other wavelengths were negative, signifying that they generally play an inhibitory role in the prediction. However, overall average trends might obscure more complex nonlinear relationships. Therefore, we calculated the specific influence patterns of each wavelength on the model’s prediction across its different values, as depicted in Figure 5b. This detailed analysis revealed that for most wavelengths, the influence is not simply positive or negative but shows complex nonlinear behavior. This indicated a non-monotonic association between the spectral signal at a given wavelength and the fat content, where its direction and magnitude of influence were highly dependent on its specific numerical value. To quantify the relative importance of each wavelength within the model, the average contribution rate was calculated based on the absolute value of its SHAP score, with the results shown in Figure 5c. The ranking revealed that model decisions were highly concentrated in a few key wavelengths: the top 9 wavelengths together account for over 80% of the total contribution, and the top 12 reach 95%.

For the PLS prediction model of protein content in foxtail millet constructed based on key wavelengths, the descriptions in Figure 5d–f are consistent with those in Figure 5a–c. The results of the comprehensive average SHAP values showed that seven wavelengths had a positive driving effect, while eight wavelengths played an inhibitory role. Regarding the contribution rates, 8 wavelengths accounted for 80% of the total contribution in the model, and 11 wavelengths reached a cumulative contribution rate of 95%.

## 4. Discussion

### 4.1. Statistical Differences in Fat and Protein Content of Foxtail Millet and Implications for Spectral Modeling

This study found significant statistical differences in the fat and protein content of foxtail millet. The average fat content (3.74%) was considerably lower than the protein content (10.43%). However, its CV (9.34%) was 2.51 times greater than that of protein (3.72%). This indicates that within the studied sample set, fat content was not only lower in absolute value but also exhibited greater variability, likely influenced by cultivar or environmental factors. In contrast, protein content remained relatively stable. This disparity likely originates from differences in their biosynthesis and accumulation mechanisms within the millet kernel. As a high-energy reserve, fat synthesis is more sensitive to factors such as the supply of photosynthetic products, temperature, and environmental conditions during the late grain-filling stage, which may lead to greater phenotypic variability [31]. In contrast, the content and composition of proteins—particularly storage proteins such as glutenins and prolamins—are more strongly regulated by genetic background, contributing to their higher stability across different samples [32]. This difference in component content and variability directly influences the difficulty and model performance of their NIR spectral detection. Theoretically, a wider concentration range and greater variability of the target component are more conducive to establishing a robust calibration model, as the model can learn the spectral response relationships across a broader concentration range. The findings of this study align with this principle. Fat content, with its wider relative distribution range, achieved superior predictive performance in its optimal model (RF) compared to the model for protein. The relatively concentrated range of protein content means its spectral differences may be partially obscured by other background factors (e.g., starch, moisture), which increases modeling difficulty and results in slightly weaker predictive capability of the model. Therefore, for the prediction of fat content, the RF model achieved an RPD_P_ of 2.219. The PLS and SVM models also attained RPD_P_ values of 1.969 and 1.950, respectively. This indicates that the RF model possesses excellent predictive capability and is suitable for direct use in practical applications. In contrast, the prediction models for protein content generally yielded lower RPD_P_ values. The PLS model achieved the highest RPD_p_ of 1.811, which is sufficient for rough estimation and can be applied to distinguish between high and low sample concentration ranges. Furthermore, models with RPD values between 1.8 and 2.0 still hold considerable potential for practical application [5]. This finding suggests that when developing non-destructive detection models for foxtail millet quality, it is crucial to fully consider the population distribution characteristics of the target components. This can be achieved by either collecting samples that cover a broader concentration range or by developing more refined spectral preprocessing and variable selection methods specifically for components with low variability, thereby enhancing the generalizability and accuracy of the models.

### 4.2. Analysis of the Advantages of SSA in Spectral Feature Extraction

Given the high-dimensional and collinear nature of near-infrared spectral data, efficient feature wavelength selection is crucial for improving model efficiency and interpretability. This study employed the SSA for feature extraction, and its advantages are demonstrated in two main aspects. First, SSA exhibits strong global optimization capability and the ability to avoid premature convergence. As shown in Figure 3a and Figure 4a, the algorithm effectively explores a vast space of wavelength combinations by simulating the foraging and anti-predation behaviors of a sparrow population. Using the MSE as the fitness function drives the search toward solutions with lower error. The 50 independent runs ensured thorough exploration of the search space, effectively preventing convergence to local optima. Consequently, this approach has a higher probability of identifying the wavelength subset that is most chemically relevant to the target component while minimizing redundancy. Second, the stability screening strategy based on frequency statistics is another key advantage. This study innovatively performed statistical analysis on the frequency with which each wavelength was selected across the 50 runs, as shown in Figure 3b and Figure 4b. A high occurrence frequency (≥50%) was used as a key indicator of stable relevance. This strategy effectively distinguishes between wavelengths selected by chance and those of stable importance, filtering out noise introduced by the algorithm’s randomness and thereby refining the selection to feature wavelengths with genuine physico-chemical significance. Ultimately, the number of wavelengths for fat and protein was reduced to 13 and 15 key wavelengths, respectively, accounting for only 8.784% and 10.135% of the full spectral bands. This significantly reduced the data dimensionality. The method combining SSA with frequency analysis not only considers the correlation between wavelengths and components but also emphasizes their stability throughout multiple optimization processes, resulting in more robust outcomes.

### 4.3. Attribution Analysis of Chemical Bonds for Key Wavelength Screening Results

For the 13 key wavelengths identified for fat, they are primarily distributed across three regions. The wavelengths near 1000–1100 nm (e.g., 1003.51, 1011.22, 1048.94, and 1077.23 nm) are primarily associated with the second overtone absorptions of C–H bonds, specifically the stretching vibration overtones of methylene (–CH_2_–) and methyl (–CH_3_) groups in lipids [33,34]. The 1048 nm wavelength is often regarded as one of the characteristic absorption peaks for oil content [35,36]. The region near 1150–1250 nm (e.g., 1199.81 and 1228.10 nm) is characteristic of combination band vibrations of C–H bonds [37,38]. This is one of the most commonly used spectral ranges for detecting edible oil and fat content, reflecting the abundant information from C–H groups in fats. The wavelengths near 1350–1550 nm (1341.25, 1374.25, 1477.98, 1506.26, and 1526.84 nm) correspond to the first overtones and combination bands of C–H bonds [39,40]. Absorptions around 1210 nm and 1390 nm are associated with C–H characteristics of unsaturated fatty acids, such as oleic acid. The region of 1470–1550 nm is strongly correlated with the first overtone of the C–H stretching vibration in methylene groups of fats [41]. The wavelengths at 1289.39 and 1298.82 nm may involve combination bands related to C–H deformation vibrations. The concentrated selection of these wavelengths clearly delineates the spectral fingerprint region dominated by C–H vibrations within the molecular structure of fats. As early as 2022, Puttipipatkajorn et al. systematically outlined that the characteristic C–H absorptions for oils in oilseed crops are located around 1210 nm and 1390 nm, among other positions [42]. Similarly, Tonolini et al., utilizing hyperspectral imaging to detect fat content in soybean oil, identified sensitive wavelengths concentrated near 1210 nm and 1450 nm [43]. The key wavelengths for fat in foxtail millet identified in this study (e.g., 1199.81, 1228.10, 1477.98, and 1506.26 nm) fall precisely within these classic spectral ranges. This alignment confirms that the chemical nature of the spectral response for fat in foxtail millet is consistent with that observed in other oil-containing crops.

For the 15 key wavelengths identified for proteins, they point to different functional groups. The wavelengths near 990–1010 nm (992.363, 997.077, and 1001.79 nm) are attributed to the third overtone of the N–H stretching vibration in the amide bonds of proteins [44], as well as overtone absorptions of O–H (which may be related to protein-bound water). This region is one of the characteristic sensitive zones for proteins. The region near 1100–1200 nm (1114.94 and 1185.67 nm) is likely associated with the second overtone of N–H bonds in proteins or combination bands involving C–H and N–H vibrations [45]. The wavelengths near 1200–1400 nm (1237.53, 1270.53, 1294.10, 1341.25, 1350.68, and 1355.39 nm) cover combination bands related to the amide II band of proteins (N–H bending and C–N stretching) [44,46], as well as various combination band information from C–H bonds. This is an important spectral interval for protein quantification. In their research on wheat protein detection, Yan et al. explicitly identified key characteristic bands for protein around 980 nm, 1200 nm, and in the 1470–1550 nm and 1650–1680 nm ranges [47]. The wavelengths found in this study for foxtail millet—992–1001 nm, 1185 nm, 1440–1449 nm, and 1647 nm—closely correspond to these regions. Similarly, Ye et al., in their study on barley protein, emphasized the importance of the 1100–1150 nm and 1300–1350 nm regions, which aligns with the 1114.94 nm wavelength and the 1341–1355 nm range identified in the current study [48]. This consistency across different crops demonstrates that the characteristic absorptions in the near-infrared region, arising from vibrations of amide bonds (N–H, C=O, C–N) in proteins, are universal. This study successfully captured these core fingerprint features in foxtail millet. It is noteworthy that the wavelength 1341.25 nm appears in both the key wavelength sets for fat and protein, which precisely reflects the complexity of near-infrared spectroscopy. This band is likely an overlapping region of C–H and N–H vibrations, indicating that the spectral information of fat and protein in foxtail millet shares certain collinear regions.

### 4.4. Decision Mechanism Interpretation Based on SHAP

The SHAP analysis of the fat RF model revealed several key insights. First, only two wavelengths, 1374.25 nm and 1506.26 nm, exhibited a positive driving effect on the predictions. This aligns with their chemical interpretation, as they are located within strong C–H characteristic absorption regions for fat (particularly around 1506 nm, which is strongly associated with methylene C–H groups). A decrease in reflectance at these wavelengths typically corresponds to an increase in fat content. In contrast, the remaining wavelengths showed an overall negative influence. This is likely because these spectral bands are also strongly interfered with by background components such as protein and starch, resulting in a negative correlation or more complex competitive relationship between their reflectance changes and fat content. Second, the vast majority of wavelengths exhibited non-monotonic SHAP dependence. This means that for a single wavelength, the direction and magnitude of its influence on the predicted value can change depending on its specific reflectance value. This confirms the RF model’s capability in handling complex nonlinear relationships and suggests that simple linear regression might be insufficient to fully capture these intricate interactions. Third, the contribution to the model’s predictions was highly concentrated. The top 12 wavelengths accounted for 95% of the predictive power. Notably, the wavelengths with the highest contributions (e.g., 1199.81 and 1228.10 nm) are precisely within the core region for C–H combination bands in fats. This demonstrates that the key wavelength set selected by SSA is not only concise but also forms the absolute core basis for the model’s decision-making.

For the protein PLS model, while PLS itself possesses inherent interpretability through latent variables, the SHAP values provide local, instance-level explanations. The results indicate both positive and negative directional influences, and the contribution is similarly concentrated in a small number of wavelengths (the top 11 account for 95%), with particularly prominent contributions from the wavelength 1647.71 nm (amide I band) and those in the 990–1010 nm range (N–H overtones). This, from another perspective, validates the fundamental importance of these wavelengths for protein prediction.

### 4.5. Strengths and Limitations of This Study

The primary strengths of this study lie in its systematic methodological integration and interpretability-driven approach. Firstly, it integrates the SSA with a frequency statistics strategy for robust feature wavelength selection in near-infrared spectroscopy. This combination and its application within a stability-analysis framework enhance the global optimization capability of the selection process and improve the robustness of the results, achieving effective data dimensionality reduction with clear physicochemical grounding. Secondly, beyond constructing quantitative prediction models, the study incorporates SHAP values to provide post-hoc interpretability of the model decisions, thereby improving the transparency of the machine learning pipeline for this application. Thirdly, by conducting a detailed chemical bond attribution analysis for the key wavelengths identified by SSA, the study directly links spectral features to the molecular structures of fat and protein, which strengthens the mechanistic foundation and supports the broader relevance of the findings.

This study also has several limitations, which help appropriately contextualize its findings. The primary limitation lies in the genetic and geographical homogeneity of the sample set. All millet samples were sourced from a single ecological region and consisted of only one cultivar. While this helped control for extraneous variables, it limits the generalizability of the calibration model to a wider range of cultivars, growing environments, and potential genotype-by-environment interactions. Secondly, although the sample size was sufficient for preliminary modeling, it may not fully capture the extreme compositional variations encountered in actual production, which could affect the model’s robustness in broader applications. Thirdly, while the SSA identified key wavelengths, the inherent spectral overlap and collinearity between fat and protein information in the NIR region may impose certain constraints on the model’s specificity and accuracy. Fourthly, regarding model validation strategy, this study primarily relied on a single random data split for final performance evaluation, which may lead to an insufficient assessment of model robustness. Fifthly, this study has not yet explicitly defined the Applicability Domain for the developed predictive models. Additionally, the research did not delve into the effects of variations in sample physical states (such as particle size and moisture content) on spectra and model performance. Therefore, future studies should employ more diverse and larger sample sets, coupled with more rigorous validation strategies and uncertainty analysis, to develop and validate non-destructive detection models with greater generalizability and metrological reliability.

## 5. Conclusions

This study conducted a systematic analysis of the fat and protein content in 214 foxtail millet samples. Combined with near-infrared spectroscopy, it provided an in-depth investigation into the characteristics of and differences between the two components regarding their content distribution, spectral response, and predictive modeling. The analysis revealed that both fat and protein content exhibited relatively concentrated distribution trends. Protein content was higher and showed less variability among samples, while fat content demonstrated a moderate degree of dispersion. The characteristic absorptions in specific spectral bands were closely related to the molecular structures of fat and protein, and the application of S-G and SNV preprocessing effectively enhanced the quality of the spectral signals.

Key wavelength screening based on SSA significantly reduced data dimensionality, identifying 13 and 15 characteristic wavelengths highly associated with fat and protein content, respectively. Model construction results indicated that the RF model performed best for fat content prediction, whereas the PLS model was optimal for protein content prediction. This suggests differences in the underlying association mechanisms between each component and the spectral data. Furthermore, by employing the SHAP method for model interpretation, it was found that the influence of key wavelengths on prediction outcomes exhibited significant nonlinear characteristics. Model decisions were primarily concentrated on a small number of important wavelengths, enhancing the model’s credibility and interpretability.

In summary, this study established a comprehensive, rapid detection methodology for foxtail millet quality, integrating statistical analysis, spectral preprocessing, feature selection, and machine learning. It not only clarified the distribution patterns of fat and protein content but also provided a reliable technical pathway for the efficient, non-destructive detection of these components in millet. This work offers a scientific foundation for subsequent endeavors in portable device development, quality evaluation, cultivar breeding, and processing utilization.

## Figures and Tables

**Table 1:** Results of detection models for fat and protein content (%) in foxtail millet.

Type	Model	Training Set			Prediction Set		
		R_C_^2^	RMSE_C_ (%)	RPD_C_	R_P_^2^	RMSE_P_ (%)	RPD_P_
Fat	PLS	0.772	0.226	2.184	0.742	0.247	1.969
	RF	0.806	0.210	2.270	0.797	0.218	2.219
	SVM	0.791	0.220	2.187	0.737	0.253	1.950
Protein	PLS	0.734	0.247	1.939	0.695	0.268	1.811
	RF	0.492	0.320	1.403	0.472	0.328	1.376
	SVM	0.593	0.296	1.567	0.538	0.314	1.471

## Data Availability

The original contributions presented in this study are included in the article. Further inquiries can be directed to the corresponding authors.

## Abbreviations

The following abbreviations are used in this manuscript:

NIR	Near-Infrared Spectroscopy
SSA	Sparrow Search Algorithm
PLS	Partial Least Squares Regression
RF	Random Forest
SVM	Support Vector Machine
SHAP	SHapley Additive exPlanations
DNN	Deep Neural Network
CNN	Convolutional Neural Network
S–G	Savitzky-Golay
SNV	Standard Normal Variate
MSE	Mean Squared Error
CV	Coefficient of Variation
R^2^	Correlation coefficient
RMSE	Root mean square error
RPD	Relative Percent Deviation
